# Unravelling the Evolutionary Complexity of Orf Virus: A Global and Multi-Host Perspective

**DOI:** 10.3390/v18020222

**Published:** 2026-02-10

**Authors:** Giada Lostia, Chiara Locci, Angela Maria Rocchigiani, Carla Cacciotto, Mariangela Stefania Fiori, Ilenia Azzena, Yoel Rodriguez-Valera, Alessandra Mistral De Pascali, Martina Brandolini, Davide Pintus, Ciriaco Ligios, Alessandra Scagliarini, Marco Casu, Elisabetta Coradduzza, Fabio Scarpa, Daria Sanna, Giantonella Puggioni

**Affiliations:** 1 Istituto Zooprofilattico Sperimentale della Sardegna, 07100 Sassari, Italy; giada.lostia@izs-sardegna.it (G.L.); angelamaria.rocchigiani@izs-sardegna.it (A.M.R.); mariangela.fiori@izs-sardegna.it (M.S.F.); davide.pintus@izs-sardegna.it (D.P.); ciriaco.ligios@izs-sardegna.it (C.L.); giantonella.puggioni@izs-sardegna.it (G.P.); 2 Dipartimento di Medicina Veterinaria, University of Sassari, 07100 Sassari, Italy; c.locci3@phd.uniss.it (C.L.); ccacciotto@uniss.it (C.C.); iazzena@uniss.it (I.A.); marcasu@uniss.it (M.C.); 3 Dipartimento di Scienze Biomediche, University of Sassari, 07100 Sassari, Italy; fscarpa@uniss.it; 4 Mediterranean Center for Disease Control, University of Sassari, 07100 Sassari, Italy; 5 Faculty of Agricultural Sciences, University of Granma, Bayamo 95100, Cuba; yrodriguezvalera@udg.co.cu; 6 Dipartimento di Scienze Mediche e Chirurgiche, University of Bologna, 40138 Bologna, Italy; alessandra.depascal3@unibo.it (A.M.D.P.); martina.brandolini3@unibo.it (M.B.); alessand.scagliarini@unibo.it (A.S.)

**Keywords:** ORFV, phylodynamics, VIR gene, accidental hosts, *Parapoxvirus*

## Abstract

Orf virus (ORFV), a member of the *Parapoxvirus* genus, is commonly associated with a highly infectious skin disease primarily affecting sheep and goats, with a reported zoonotic potential. Initially identified in the 18th century, ORFV has been sporadically reported in other species, including humans. The present study analyzed the genetic variability and phylodynamic patterns of ORFV using the highly variable VIR gene, focusing on global strains from multiple hosts, including various species of ruminants and humans. A dataset of 267 ORFV strains from around the world, including sequences from the understudied island of Cuba, was used for the analyses. Results revealed greater genetic variability for ORFV than previously reported. While the virus may be defined as a generalist pathogen, capable of infecting various ruminant species and less frequently humans, signs of host-specific specialization are emerging exclusively for sheep and goats. Other ruminant species and humans may be categorized as occasional hosts, with infections likely linked to habitat overlap with sheep and goats and sporadic transmission that appears influenced by specific risk factors. In conclusion, these findings contribute to a better understanding of the transmission risks posed by ORFV, highlighting the need for further investigations into its potential to infect a broader range of hosts, particularly humans.

## 1. Introduction

Orf, also referred to as contagious pustular dermatitis (CPD) or contagious ecthyma (CE), is a highly infectious viral skin disease [1] with a worldwide distribution [2]. Initially identified in sheep by Steeb in 1787 and later observed in goats and humans by Hansen in 1879 [3], the disease is caused by the Orf virus (ORFV), a member of the *Parapoxvirus* genus, within the Chordopoxviridae subfamily of the Poxviridae family [4].

In this context, contagious ecthyma has been reported worldwide, with documented cases across Europe (Greece, Norway, Italy, England, Romania, Bulgaria, Hungary), North America (USA), South America (Brazil, Argentina), Asia (India, Taiwan, China, Israel, Japan, Iran, Iraq, Malaysia, Korea, Turkey, Lao, Bangladesh, Mongolia, Saudi Arabia, Pakistan), Africa (Nigeria, Kenya, Sudan, Zambia, Gabon, South Africa, Cameroon, Egypt, Ethiopia, Morocco), and Australia [4,5,6].

Outbreaks of CPD most frequently occur between autumn and spring months [7], with the highest severity observed during the autumn and winter months [8,9,10], particularly in December and January in the Northern Hemisphere, as reported by Robinson et al. (1983) [7]. However, sporadic cases have also been documented during the summer months [11,12,13].

Although ORFV is commonly associated with a skin disease affecting sheep and goats [14], it has also been reported sporadically in other ruminants and non-ruminant species [15]. With regard to other ruminants’ species, several studies have reported ORFV in members of the Bovidae family, including domestic cattle [16], free-ranging and captive musk ox [17,18,19,20], captive Sichuan takin [19], and wild chamois [21]. Cases have also been documented in the Antilocapridae family, such as in wild Japanese serow [22], and wild ibex [23], as well as in the Cervidae family, specifically in reindeer [24,25,26].

With regard to non-ruminant species, ORFV has been reported in member of the Camelidae family, including domestic dromedaries [27,28,29,30], cats [31], and humans [5,32,33]. Finally, Cargnelutti et al. [34] reported that mice and rabbits became susceptible to ORFV when inoculated with the IA-82 ORFV strain via skin scarification at various anatomical sites under experimental conditions. Notably, the infected rabbits developed skin lesions that closely resembled those observed in both naturally and experimentally infected sheep [34].

It is important to highlight that ORFV is considered to have a zoonotic potential in humans [35]. The transmission usually occurs through direct contact (through abrasion or breaks in the skin) with infected animals, or through contaminated fomites [7,36,37]. Individuals who work closely with animals (e.g., shepherds, farmers, and veterinarians) are considered to be at higher risk of infection [7]. Additionally, a case report has described ORFV transmission to a 53-year-old woman with psoriasis, living in a farming community, after she was scratched by a stray cat [38]. This extensive host range contributes to the virus’s endemicity in areas where animals are abundant, suggesting a persistent opportunity for dynamic interaction between the virus and its hosts [39].

The virus causes persistent and localized skin lesions, that typically last between three and four weeks [40]. It is also capable of reinfection, due to its ability to evade the host’s immune response [41]. In animals, lesions commonly develop on the lips, muzzle, ears, eyelids, and the nasal mucosa, and, less frequently, on the mammary glands, genitals, and feet [15,42]. In humans, lesions are often located on exposed areas of the skin, such as the hands, fingers, and forearms, which are most likely to come into contact with animals [43,44].

The virus primarily affects young animals, including lambs and kids, although adult animals can also be infected [41]. The disease has been reported to occur irrespective of age, sex, or breed [15]. During ORFV outbreaks, the morbidity rate is generally higher than the mortality rate [35]. However, young animals (e.g., lambs), which are more susceptible to the disease [15], experience higher mortality rate compared to adults [41]. The disease usually results in death due to the presence of painful lesions around the mouth that prevent suckling, maternal rejection because of severe udder lesions, or complications arising from secondary bacterial or fungal infections and maggot infestation [41].

Research efforts have focused on exploring the genetic diversity and evolutionary history of ORF virus and providing insights into the potential geographic clustering of circulating strains [45].

Traditionally, the largest number of studies have focused on variation at single-gene loci (e.g., [46,47,48]), with particular attention on the gene VIR (encoding for viral interferon resistant). This gene has widely been investigated to infer the phylogeography and evolutionary dynamics of ORFV (e.g., [48,49,50,51,52,53,54,55,56,57]). VIR is considered a major virulence factor of ORFV and represents a variable genomic region within the *Parapoxvirus* genus [51,56]. Although other ORFV genes (e.g., orf109, orf110, orf132) show higher levels of nucleotide variability [46,47,58], they are comparatively less represented in the literature, limiting the use of their sequences in large-scale phylogenetic and comparative analyses. Within this framework, phylogeographic studies based on the analysis of VIR variation reported that it is highly informative for genetic characterization within *Parapoxvirus*. Notably, VIR has proven to be an efficient indicator of genetic variability and population structure in ORFV [48,50,52,55,56,57]. Several studies across this locus have indicated the potential existence of host-associated specific strains for sheep and goats [50,57,59]. However, it should be taken into consideration that this gene was reported to exhibit conservation in amino acid residues, and this pattern is not associated with phylogenetic groupings based on host species, geographical origin, or time of isolation [52].

In addition to VIR, other ORFV genes, such as B2L (gene encoding for the immunogenic major envelope protein) and O45 (gene encoding for the late transcription factor VTLF-1), have been employed in genetic analyses aimed at clarifying the evolutionary history of this virus. Although results obtained from these loci are generally consistent with those based on VIR, their corresponding indices of genetic variation and informativeness are generally lower (e.g., [50,55,56,57,59]).

More recently, research has moved towards genomic-scale analysis. In particular, based on the limited number of whole genomes available to date [46,49,50,60,61,62,63,64], Coradduzza et al. [50] reported that modern sheep and goat ORFV lineages diverged from one another in the second half of the 20th century, originating from two geographically distinct clusters. Their study also hypothesized the possible existence of additional viral strains capable of infecting either goats or sheep.

In the present study, the ORFV molecular marker for which the largest number of sequences is currently available in the literature was analyzed. For this reason, the highly variable VIR gene was employed to analyze the genetic diversity of ORFV on a global scale, across natural, occasional or accidental, and human hosts, using the most comprehensive approach applied to date. Specifically, VIR gene has been used to investigate in detail the phylodynamic patterns of the virus and to explore early signs of host-specific adaptation in sheep and goats, based on an expanded dataset that includes understudied geographical areas and strains isolated from various host species. Furthermore, 31 sheep and goat samples collected from an under-investigated area of the island of Cuba were combined with a dataset comprising VIR sequences available in GenBank, contributing to a more comprehensive assessment of the global diversity and evolutionary dynamics of ORFV.

## 2. Materials and Methods

### 2.1. Sampling

The Cuban new pathological samples in this study were collected between 2005 and 2009 (as summarized in Table 1) from sheep and goats naturally infected by the disease during various outbreaks in the municipalities of Holguin, Granma, Santiago De Cuba, and Guantanamo of the Eastern Provinces of Cuba (Supplementary Figure S1).

**Table 1:** Table providing the information on the 31 Cuban strains analyzed and their related GenBank accession numbers (GB#).

Sample ID	GB#	Collection Site	Collection Date	Host
S1	OR338563	Santiago de Cuba	21 March 2006	sheep
S2	OR338564	Santiago de Cuba	22 March 2006	sheep
S3	OR338565	Guantanamo	7 March 2006	sheep
S8	OR338566	Guantanamo	22 June 2006	sheep
S46	OR338567	Guantanamo	17 April 2008	goat
S48	OR338568	Guantanamo	29 December 2008	sheep
S18	OR338569	Granma	15 June 2006	goat
S21	OR338570	Guantanamo	18 July 2006	goat
S23	OR338571	Guantanamo	25 September 2006	sheep
S37	OR338572	Guantanamo	17 April 2008	goat
S40	OR338573	Guantanamo	25 April 2008	goat
S42	OR338574	Guantanamo	28 July 2008	sheep
S835-3	OR338575	Granma	3 June 2005	goat
S28	OR338576	Holguin	14 September 2005	sheep
S30	OR338577	Granma	4 June 2006	goat
S5	OR338578	Guantanamo	5 March 2006	sheep
S44	OR338579	Granma	25 December 2008	sheep
S51	OR338580	Holguin	5 June 2009	sheep
S10	OR338581	Guantanamo	23 June 2006	goat
S15	OR338582	Granma	4 June 2006	sheep
S31	OR338583	Guantanamo	9 June 2007	sheep
S33	OR338584	Guantanamo	10 November 2007	goat
S34	OR338585	Guantanamo	1 February 2008	goat
S36	OR338586	Guantanamo	14 January 2008	sheep
S38	OR338587	Guantanamo	13 November 2008	goat
S39	OR338588	Guantanamo	10 October 2008	sheep
S41	OR338589	Guantanamo	29 December 2008	goat
S43	OR338590	Guantanamo	17 January 2009	sheep
S45	OR338591	Guantanamo	10 October 2008	sheep
S50	OR338592	Holguin	5 June 2009	sheep
S49	OR338593	Santiago de Cuba	10 April 2009	sheep

To obtain a comprehensive dataset representing all viral sequences available worldwide from several host species, the ORF sequences obtained from these Cuban samples (refer to Table 1 for accession number) were merged with available VIR gene sequences deposited in GenBank (see Supplementary Table S1 for details). The resulting dataset included a total of 267 strains from 21 countries and 5 continents, belonging to 6 different hosts, with the exception of 20 strains for which the host species of origin was not indicated (14) or specified accurately (6 referred as “small ruminants”).

### 2.2. Viral DNA Extraction

To confirm the presence of ORFV, DNA was extracted from 25 mg of each lesion tissue sample using the DNeasy Blood and Tissue Kit (Qiagen, Germantown, MD, USA) according to the manufacturer’s instructions. Then, VIR was subjected to amplification using the VIR1 and VIR2 primers [19]. This process provided the generation of fragments measuring 617 bp. To amplify VIR from samples negative to the first PCR, an additional primer set (VIR 3 and VIR 4) was designed, which amplifies a fragment of approximately 817 bp. The PCR was performed as previously described by Kottaridi et al. [52] with slight changes in annealing temperatures [52]. Positive (previously characterized high quality ORFV DNA samples from reference collection) and negative controls were used to evaluate the efficacy of PCR protocols and to ascertain the absence of any potential inhibitors or contaminants. Electrophoreses were carried out with Invitrogen E-Gel EX 2% agarose kit (Carlsbad, CA, USA) (gel precast). Specific DNA PCR bands were excised from the gel and purified using the QIAquick Gel Extraction Kit (Qiagen). When non-specific bands were also present during electrophoresis, PCR products were purified using the CleanSweep PCR Purification kit (Thermo Fisher Scientific, Waltham, MA, USA).

### 2.3. Sequencing

All reliable PCR products were then subjected to sequencing for both the forward and reverse strands, using the same primers utilized for the PCR. In detail, the viral DNA was quantified using an Epoch microplate spectrophotometer (BioTek, Winooski, VT, USA) and a Qubit 2.0 Fluorometer (Thermo Fisher Scientific) according to the manufacturers’ instructions. DNA viral libraries were then prepared using the Nextera DNA Flex Library Prep kit (Illumina Inc., San Diego, CA, USA). The following whole-genome sequencing was performed by BMR Genomics (an external core service; BMR Genomics, Padua, Italy) using the whole-genome shotgun technique (WGS) on the MiSeq platform (Illumina) to generate paired-end reads of 2 × 300 bp.

### 2.4. Phylogenetic Analysis

A total of 31 VIR gene sequences were obtained in the present study.

Noteworthy, to maximize the dataset, we utilized a 382 bp VIR gene fragment, enabling the inclusion of all available GenBank sequences regardless for their size. Retaining a comprehensive library representative of all host species was critical for ensuring the reliability of results.

After checking sequences using Unipro UGENE 35 [65], they were aligned with the Clustal Omega 1.2.4 software package [66] available at https://www.ebi.ac.uk/Tools/msa/clustalo/ [67] (accessed on 24 August 2025). The sequences have been submitted to the GenBank online database, and their accession numbers are provided in Supplementary Table S1.

To place our data within a broader geographic framework, we built a dataset that included the 31 VIR gene sequences obtained from Cuban samples along with the publicly available ORF VIR gene sequences deposited in GenBank. This extensive dataset encompassed sequences from across all continents, comprising Asia (145), Europe (57), South America (21), North America (41), Oceania (2), and Africa (1). Only sequences that perfectly overlapped with the VIR gene fragment analyzed in this study were included, resulting in a final dataset of 267 sequences used for further analyses. Additionally, a Pseudocowpox virus sequence (GQ329670) was used as outgroup.

Notably, a total of 252 ORF VIR sequences out of the 267 available in the dataset could be assigned to specific host species. In particular, 247 have been isolated from ruminants (136 sequences belonged to goats, 93 to sheep, 10 to Japanese goat-antelopes, 6 to small ruminants, 1 to Sichuan takin, 1 to musk ox), and 5 from humans.

To evaluate genetic variation across the whole dataset and within and among host-associated populations, the number of polymorphic sites (S), number of haplotypes (H), nucleotide diversity (π), and haplotype diversity (h), have been estimated by means of the software DnaSP 6.12.03 [68].

The best probabilistic model of sequence evolution was identified using jModeltest 2.1.1 [69], applying a maximum likelihood optimized search by the Akaike (AIC) and Bayesian Information Criterion (BIC). Both criteria consistently identified GTR + I + G as the best-fitting model.

To assess the suitability of the VIR gene dataset for phylogenetic and phylodynamic analyses, the phylogenetic signal [70] was evaluated through a likelihood mapping of 10,000 randomly selected quartets, conducted using TreePuzzle 5.3 [71].

A Bayesian phylogenetic tree was constructed using MrBayes 3.2.7 [72], applying the following model parameters: setting nst = 3, rates = gamma, and ngammacat = 4. Two separate runs were performed simultaneously, each involving 4 Metropolis-coupled Markov chain Monte Carlo (MCMC) chains (1 cold and 3 heated). The MCMC process was run for 5 million generations, with trees sampled every1000 generations. The initial 25% of sampled trees were excluded as burn-in. Analyses were carried out through the CIPRES Science Gateway platform [73]. To confirm convergence of chains, the average standard deviation of split frequencies (expected to approach) [66] and the potential scale reduction factor (expected to be approximately close to 1) [74], have been assessed following the guidelines described by Scarpa et al. [75]. The resulting tree was then visualized and edited using FigTree 1.4.0 [76] (available at http://tree.bio.ed.ac.uk/software/figtree/, accessed on 22 December 2025).

Molecular dating was conducted using a Bayesian approach under the MCMC algorithm implemented in Beast 1.10.4 software [77], incorporating the sampling dates of the sequences. To determine the best molecular clock model, both strict and uncorrelated log-normal relaxed clocks were evaluated through preliminary runs of 100 million generations. Model selection was guided by the comparison of Bayes factor values using Tracer 1.7 [78]. All available demographic models, both parametric and non-parametric, were also evaluated to determine the best fit for the sequence data. Following model selection, time-calibrated phylogenetic trees and evolutionary rates were co-estimated for the dataset by running the analysis for 300 million generations, sampling every 30,000 generations. The output log files were assessed in Tracer 1.7 software [79], retaining only those with ESS (effective sample size) values above 200. The maximum clade credibility tree was then generated using TreeAnnotator (Beast package) and subsequently visualized and annotated in FigTree 1.4.4.

Finally, Principal Coordinate Analysis (PCoA) was performed in GenAlEx 6.5 [80] to explore genetic variation and identify potential subgrouping within clusters. The analysis relied on a pairwise p-distance matrix to represent genetic dissimilarity among sequences. PCoA was first conducted on the full dataset and then repeated on subsets identified from patterns observed in the initial run.

## 3. Results

### Phylogenetic and Phylodynamic Analyses

Genetic variation analysis conducted on the whole dataset of 267 sequences identified 41 polymorphic sites, resulting in 44 VIR haplotypes (h = 0.924; π = 0.0423). Overall, the dataset showed high levels of genetic diversity. A subset of 252 sequences, including only strains to which it has been possible to assign a specific host species, was used to evaluate genetic diversity within and among host-associated strains (see Supplementary Table S2: 136 goats, 93 sheep, 10 Japanese goat-antelopes, 6 small ruminants, 1 Sichuan takin, 1 musk ox, and 5 humans).

Overall, for this subset of 252 sequences, genetic diversity remained high (h = 0.932; π = 0.0418) with 43 haplotypes, thus reflecting the high genetic variability present among the host-associated lineages.

Within this subset, the 247 ruminant-associated sequences (excluding the 5 human isolates) also exhibited high overall genetic diversity, with 43 haplotypes detected (h = 0.931; π = 0.0419). In particular, goats and sheep showed the highest levels of genetic diversity. Goats displayed 70 haplotypes among 136 sequences (h = 0.978; π = 0.0361), while sheep showed 54 haplotypes among 93 sequences (h = 0.971; π = 0.0366), both indicating high within-host genetic variation. On the other hand, the occasional hosts, which included Japanese-goat antelope, small ruminants, musk ox, Sichuan takin, and human ORFV strains, exhibited lower levels of genetic diversity (see Supplementary Table S2 for details on host species groups), with 10 haplotypes among 23 sequences (h: 0.783; π = 0.0266). Within the occasional hosts group, the 5 human-associated sequences were particularly differentiated among each other: each of the 5 human isolates presented distinct private haplotypes/strains (h = 1), with nucleotide diversity values (π = 0.0377) comparable to that observed for sheep and goat lineages. Notably, polymorphic sites characterizing human lineages included both neutral and missense mutations, and all the resulting amino acid changes were also found for ruminant-associated polymorphic strains.

The likelihood-mapping analysis performed on the complete dataset of 267 sequences (Supplementary Figure S2) revealed a robust phylogenetic signal, with 2.8% of unresolved quartets, significantly under the 30% threshold commonly indicative of poor phylogenetic resolution [69].

**Figure A1:**
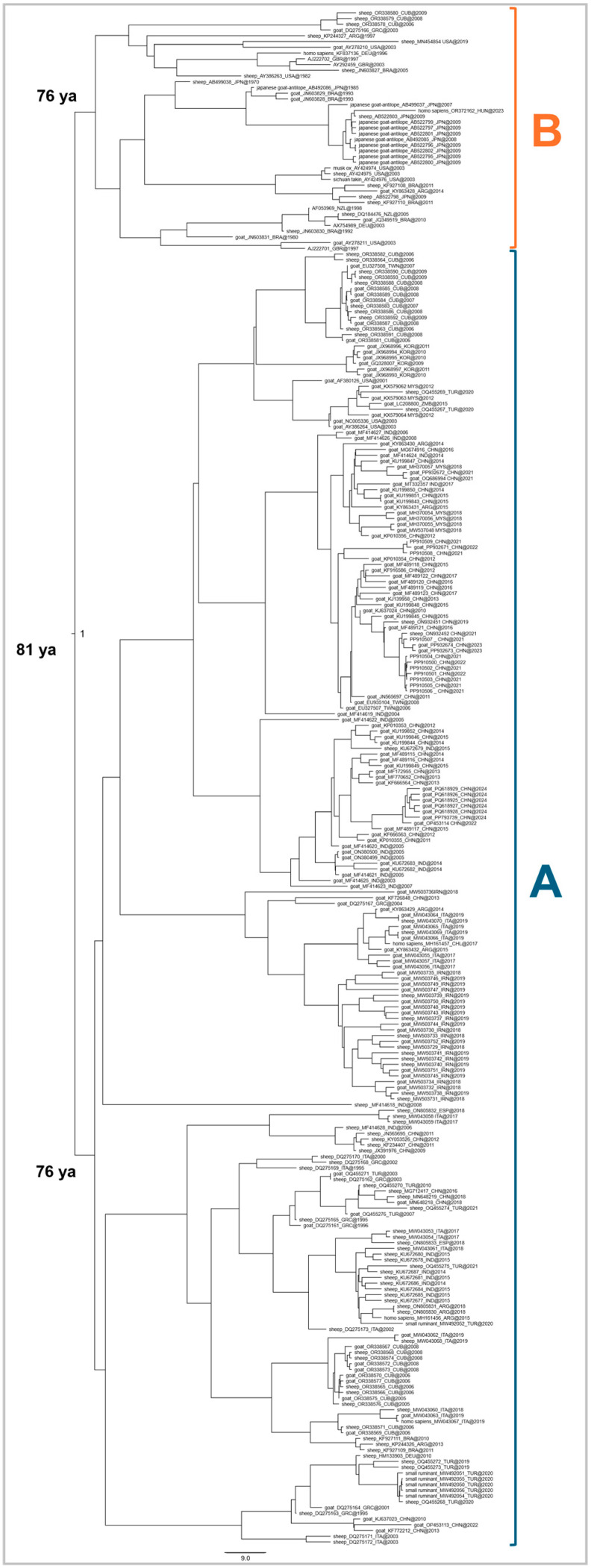
Bayesian phylogenetic tree of the whole dataset with divergence time estimates at major nodes obtained by the software Beast 1.10.4. Group subdivisions are described in the text. Posterior probability values are indicated at the basal node (*pp* = 1.0).

The Bayesian phylogenetic tree, also conducted on the whole dataset of 267 sequences, was consistent with the topology of the ultrametric tree. Based on this concordance, a graphical representation of the Bayesian tree, incorporating divergence times at major nodes, was generated (see in Appendix A, Figure A1). Phylogenetic reconstruction revealed the presence of two principal genetic clusters, designated as A and B, strongly supported at their basal node (*pp* = 1.0). These clusters diverged from a common ancestor which dated back to approximately 1943 (81 years before 2024, the year in which the most recent strain was collected) and appeared to have emerged contemporaneously, around 1948 (76 years before 2024). Cluster A included the majority of sequences in the dataset, comprising viral strains isolated from sheep, goats, humans, and small ruminants, collected worldwide between 1995 and 2024. Several sub-clusters were detected; however, most of these subdivisions were weakly supported (*pp* < 0.90), suggesting limited phylogenetic resolution. A few sub-groups exceeded the support threshold (*pp* > 0.90), but did not show structuring by geography, collection date, or host species. Cluster B contained fewer sequences, primarily from goats and sheep, along with a small number from humans, Japanese goat-antelopes, a musk ox, and a Sichuan takin. These isolates were collected between 1970 and 2023, mostly from North America (including most of the Cuban samples obtained in the present study), South America, Asia, and Europe. As in Cluster A, sub-structuring was observed but generally poorly supported (*pp* < 0.90).

**Figure 1:**
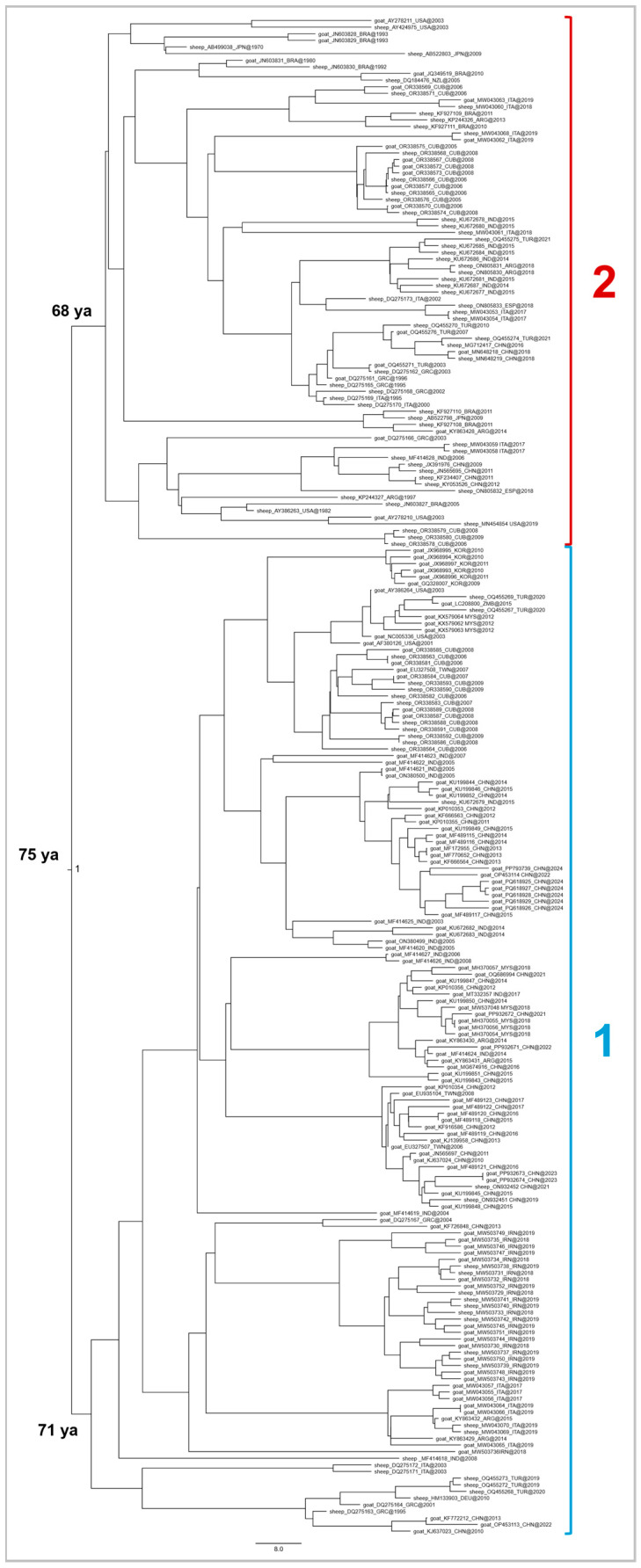
Bayesian phylogenetic tree of the sheep and goat subset with divergence time estimates at major nodes obtained by the software Beast 1.10.4. Group subdivisions are described in the text. Posterior probability values are indicated at the basal node (*pp* = 1.0).

A second Bayesian phylogenetic tree with divergence times estimates was constructed, this time including only sheep and goat isolates (n = 229) to investigate potential occurrence of genetic structuring between these two host-associated viral populations (Figure 1). The most recent common ancestor, fully supported at the main node (*pp* = 1.0), was estimated to date back to approximately 1949 (75 years before 2024). Notably, this estimation is consistent with the root divergence time of the first tree (Figure A1) including natural and accidental hosts.

From the most recent common ancestor departed two main clusters, designated as 1 and 2, which emerged almost contemporaneously. Cluster 1 diverged around 1953 (71 years before 2024) and included approximately 85% of all goat-derived sequences, mainly from Asia, with a few isolates from Europe, North and South America, and Africa, collected between 2001 and 2024. The few sheep-derived strains included within the cluster 1 accounted for 37% of all sheep-derived sequences, originating predominantly from Asia, North America and Europe, and collected between 1995 and 2021. A sub-structuring was observed but generally not strongly supported at the main nodes (*pp* < 0.90), except for one sub-group (*pp* = 0.97) containing primarily Asian goat isolates.

Cluster 2 diverged around 1956 (68 years before 2024) and comprised about 63% of all sheep-derived strains, mostly from North America and Asia, with additional sequences from Europe, South America and Oceania, collected between 1970 and 2021. The few goat-derived isolates included within the cluster 2 represented 15% of all goat-derived sequences, originating from North America and Europe, with a few from South America and Asia, and isolated between 1980 and 2019. Similar to cluster 1, sub-clusters were present but generally lacked full statistical support (*pp* < 0.90).

Principal Coordinates Analysis (PCoA) performed on the whole dataset of 267 sequences (thus excluding the outgroup) explained a cumulative variability of 75.40 (Axis 1: 43.23%, Axis 2: 20.87%, Axis 3: 11.30%, see Supplementary Figure S3 for details). In accordance with first phylogenetic tree (Figure A1), this analysis, which included various host groups, revealed low genetic structuring among most of the sequences. A central cluster showed substantial overlap, primarily comprising sequences from sheep, goats, and to a lesser extent, other ruminant species, and humans. At the margins of this main cluster, an incipient divergence was observed in a few sequences, mostly belonging to sheep and goats. One divergent strain from sheep originated from Spain (ON805832) and was isolated in 2018, while the divergent goat sequences were from China (KF916586, KU199850, MF489117, MF489118, MF489120, MF489122) and were isolated between 2012 and 2017.

**Figure 2:**
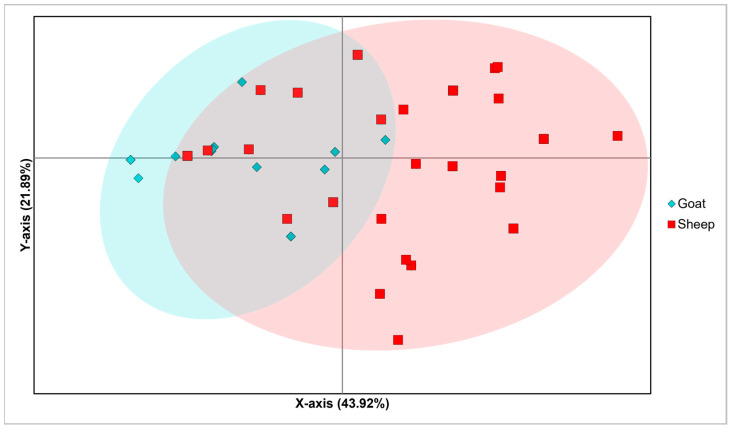
Principal Coordinates Analysis (PCoA) conducted on the subset including sequences from sheep and goats. The bi-dimensional plot displays the genetic differentiation among samples, by representing the number of nucleotide substitutions per site.

Based on these findings, a second Principal Coordinates Analysis (PCoA) was conducted on a subset, including only sequences from sheep and goats, totaling 229 sequences (Figure 2). This analysis aimed to investigate the genetic similarity between ORFV strains from these two host species. The PCoA explained a total genetic variability of 78.02% (Axis 1: 43.92%, Axis 2: 21.89%, Axis 3: 12.21%). Similar to the first PCoA (see Supplementary Figure S3), a homogeneous central cluster of overlapping for goats and sheep strains was observed, indicating low genetic differentiation among lineages. However, a slightly more pronounced divergence was evident between sequences located at the opposite x-axis extremes of the central overlapping cluster. Accordingly, a few Chinese goat sequences showed strong divergence from sheep sequences found at the opposing margins of the central overlapping area. These outlying sheep sequences originated from Argentina, India, Cuba, Turkey, Spain, and Italy.

**Figure 3:**
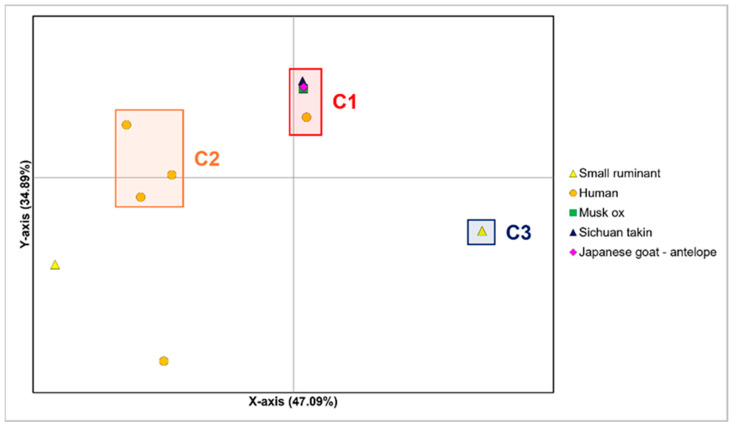
Principal Coordinates Analysis (PCoA) conducted on the subset including sequences from occasional hosts. Group subdivisions are described in the main text. The bi-dimensional plot displays the genetic differentiation among samples, by representing the number of nucleotide substitutions per site.

An additional Principal Coordinates Analysis (PCoA) was performed based on sequences from the occasional hosts, including a total of 23 isolates (Figure 3). This analysis aimed to investigate the genetic diversity and relationships among occasional hosts, comprising the Japanese goat-antelope, Sichuan-takin, musk ox, small ruminants, and human. This analysis explained a comprehensive genetic variation of 96.33% (Axis 1: 47.09%, Axis 2: 34.89%, Axis 3: 14.35%). Three main clusters, designated as C1, C2 and C3, were identified. Cluster C1 comprised the majority of sequences, including all Japanese goat-antelope isolates from Japan collected between 1985 and 2009, as well as those from Sichuan takin and musk ox, both originating from the United States and collected in 2003. This cluster also included a human-isolated strain from Hungary (OR372162—2023). Cluster C2 comprised three human-derived isolates from Germany (KF837136—1996), Italy (MW043067 –2019), and Argentina (MH161456—2015). Cluster C3 included small ruminant sequences from Turkey collected in 2020. However, one Turkish sequence (MW492052) and a human sequence from Chile (MH161457—2017) behaved as outliers, with respect to Clusters C1, C2 and C3.

## 4. Discussion

The present study explores the genetic variability and phylodynamic patterns of globally distributed Orf virus strains using a comprehensive dataset based on VIR gene variation across natural and occasional hosts. The inclusion of isolates from an under-sampled region of the island of Cuba allowed a broader evaluation of ORFV diversity at the global scale. Moreover, the VIR gene, a variable region of the ORFV genome [48,56], has proven to be highly informative for reconstructing the evolutionary history of the virus and assessing its genetic structuring.

The relevant number of sequences from a wide range of hosts used for our analyses suggests that Orf virus is a pathogen more generalist than previously thought, capable of infecting different ruminant species as well as humans (e.g., [49,50,57,81,82]). In the present study, although the virus does not exhibit a strict host specialization, as it has been suggested in previous studies (e.g., [5,25,31,41,83,84,85]), early signs of specific genetic differentiation can be observed for strains isolated from sheep and goats.

In particular, the expanded sampling of goat and sheep strains has allowed to deepen on the phylogenetic relationships among ORFV lineages, thus evidencing emerging signs of host specialization. The genetic differentiation between sheep- and goat-associated lineages has already been reported by Coradduzza et al. [49,50,57], whose divergence time estimates for the sheep and goat clusters are largely consistent with those reported in the present study. Indeed, based on complete genome data [50], the divergence of sheep and goat lineages was dated to approximately 1957 and 1947, respectively. In the present study, based on VIR gene analysis, the sheep cluster was dated to 1956 and the goat cluster to 1953. Our findings align with previous whole-genome data, further suggesting that goat strains may have diverged slightly earlier. The minor discrepancies between present and previous estimates may be attributed to the larger number of sequences analyzed in this study and to the smaller genomic region employed (382 bp for the VIR gene versus 131,449 bp for the whole genome).

Notably, consistently with Coradduzza et al. [57], that indicated VIR gene as an informative marker from a phylogenetic perspective, the results obtained here confirm that this gene broadly reflects the evolutionary dynamics of the ORF virus whole genome.

The divergence times for sheep and goat ORFV lineages differentiation correspond to the post-World War II period, a time characterized by major socio-economic and demographic changes. Indeed, historical data indicate that between 1950 and 1960 the global population of ovine species increased significantly [86]. This growth was driven by the rapid post-war agricultural recovery, marked by the development of intensive livestock breeding and a rising movement of both animals and people [87,88]. This phenomenon was accompanied by a substantial increase in the global human population beginning in the 1950s [89], which may have facilitated the spread of pathogens, including Orf virus, through the associated rise in meat consumption and the expansion of sheep and goats farming. In this context, the expansion of ovine and caprine host populations created new opportunities for viral lineages diversification and increasing of population size. The Cuban isolates fell within the major global ORFV clusters, showing genetic similarities to European and Asian strains. This suggests that historical livestock movements and human-mediated introductions shaped the viral population on the island, supporting earlier studies involving full-genome analysis [50].

Phylogenetic findings are partly supported by the PCoAs performed on both the whole dataset and the sheep-goat subset, which showed a central cluster of overlapping strains with divergence at the margins, likely reflecting the typical Wahlund’s effect [90]. This principle in population genetics describes a reduction in overall heterozygosity when a population is subdivided into smaller internal subpopulations. If a large, randomly mating population is separated into geographically isolated populations, they may develop different allele frequencies due to random genetic drift [91]. This phenomenon becomes evident when analyzing a broadly distributed population, where viral strains at the extreme edges of the host’s range begin to differentiate from each other, despite belonging to a unique, larger genetic group. In this study, the genetic variability explained by the first two axes of the PCoAs, including sheep and goat sequences, indicated that while some strains still co-infect both goats and sheep, others are undergoing divergence, suggesting ongoing host specialization.

This phenomenon may be furthered by the specific livestock management strategies adopted in different regions. Indeed, some areas are characterized by mixed sheep and goat farming, where different species share the same pastures; conversely, other contexts favor specialized production systems, which minimize the opportunities for interspecies contact. For this reason, in isolated and non-promiscuous contexts, the virus evolves by adapting to the specific local host population (either sheep or goats). Notably, the virus appears more advanced in its adaptation to goats, with only early signs of specialization in sheep.

Regarding the additional hosts included in the present study, other ruminants (different from sheep and goats) and humans appear to act as occasional hosts for ORFV, likely due to habitat sharing with sheep and goats, without establishing effective transmission among co-generics or related species. In accordance, in the phylogenetic tree most of these incidental infections are represented by isolated lineages with no descendants.

Noteworthy, in the PCoA performed on the occasional hosts group, human isolates did not show genetic affinities with the other species, including the Japanese goat-antelope, Sichuan takin and musk ox. Indeed, with the only exception of a single human-derived strain from Hungary, which clustered with the occasional hosts group, all human isolates appeared as private variants unique to individual cases, potentially reflecting the sporadic and isolated nature of these zoonotic infections. Such cases may result from rare and independent viral mutations. Human infection could therefore depend on specific risk factors that manifest only in certain individuals and under specific environmental conditions. These factors may include randomly mutated viral allelic variants, predisposing host genetic traits, immunosuppression, or environmental exposures such as direct contact with infected animals. Consequently, Orf virus infections in humans might not represent a consistent zoonotic pattern but rather sporadic, multifactorial zoonotic spillover events. A similar scenario is observed with the cowpox virus, another member of the Poxviridae family, which has expanding animal hosts, but it causes rare human infections. The severity of cowpox virus in humans depends on the host’s immune status, skin integrity, and environmental exposure [92,93]. Future research should aim to better disentangle the mechanisms that drive human infection and focus on genome-wide association analyses integrating viral genomic data with human genetic, immunological, and environmental conditions. The availability of additional complete human Orf virus genomes would also be essential to identify potential viral allelic variants associated with specific environmental or host factors.

In this context, the broader dataset used in the present study has also revealed a complex network of strains, including potential recombinant or mutated unique lineages that fail to persist over time and spread among a large number of hosts. This perspective challenges the strong host-specific structure reported in earlier studies, which were based on a smaller number of sequences from sheep, goats, and a few human isolates [52,53]. Those findings may have been biased by reduced sampling, overlooking the virus’s ability to infect occasional hosts without sustaining transmission.

Two possible scenarios may explain the presence of ORFV strains in occasional hosts. First, occasional hosts might become infected with viral variants that arise from random nucleotide mutations, enabling the infection of new species. These events are likely sporadic and stochastic, not driven by selection, whose effects are probably destined to disappear rapidly. Second, recombination events may occur within co-infected hosts, generating unique variants that disappear with the host’s death and are not transmitted further.

Studying these strains, isolated from occasional hosts, is crucial for understanding the genome evolutionary dynamics of ORFV and determining whether such lineages are exclusive to single individuals or restricted to specific ruminant populations which have been in contact with primary hosts.

Notably, both scenarios may plausibly explain the origin of the human isolated variants private to single individuals. Interestingly, for these human-derived variants, mutations facilitating spillover may have occurred in genomic regions other than the VIR gene. Indeed, the polymorphisms observed for this gene in humans do not result in human-specific amino acid changes but rather correspond to those found in sheep and goat lineages that likely spread among ovine and caprine that live in the same areas.

Further investigations into the genetic variability of the Orf virus, using either complete genomes or polymorphic genes such as VIR, are crucial to better understanding its capacity to infect a broader range of hosts beyond sheep and goats, with a particular focus on human infections.

Such research would be essential for assessing the likelihood of future cross-species transmission and spillover events. The findings may contribute to a more accurate evaluation of the zoonotic risk posed by Orf virus, particularly regarding the potential that its occasional transmission to humans and other mammal species could increase as a result of a possible enhanced infective potential in non-ovine and non-caprine hosts.

## 5. Conclusions

In conclusion, our results evidence that the VIR gene of Orf virus exhibits higher genetic variability than previously reported [50,57]. The findings highlight the virus ability to infect several ruminant species and humans as occasional hosts, without enabling transmission within those populations. Based on these outputs, we hypothesize the existence of three main genetic groups of lineages for this virus: two host-specific groups (one for goats and one for sheep), showing early genetic differentiation among each other, and a third generalist group, still capable of infecting elective hosts (sheep and goats), but also diffused in other ruminant host species and humans.

Interestingly, the results obtained in the present study suggest that the dynamics of ORFV transmission to humans may be more sporadic than traditionally assumed.

Although ORFV is commonly considered as a zoonotic pathogen, this classification generally assumes that the animal host acts as a reservoir and that the pathogen can efficiently cross the species barrier under natural conditions, even if the frequency of transmission may vary. In contrast, the present findings indicate that ORFV transmission from sheep and goats may require a combination of uncommon and highly specific circumstances (e.g., a particular viral mutation, a rare human genetic susceptibility, and intense exposure). Furthermore, transmission may occasionally arise from co-infection of the same individual with different viral strains, enabling recombination events. Under these conditions, this virus may not behave as a typical, efficiently transmitted zoonosis and human infection appears to be a dead-end spillover event rather than the result of viral adaptation to the human host. However, it should be taken into consideration that human infections in immunocompetent individuals are rarely reported in endemic areas, as the disease is generally considered self-limiting and requires no medical intervention. Consequently, this leads to a limited number of isolated viral strains from human hosts, which may slightly bias the present results.

In conclusion, these new findings suggest that ORFV may have a limited capacity for effective spillover and replication in human cells. The requirement for specific stochastic viral mutation or recombination events indicates that the general viral genome structure is not naturally adapted to human hosts. Furthermore, the species barrier between sheep and goats and humans appears to remain intact, with transmission occurring only when several unlikely conditions coincide. From this perspective, human infection may arise not from adaptation driven by natural selection, but from stochastic events facilitated by sporadic nucleotide mutations that provide no advantage to either the virus or the host and thus do not persist over time. In this context, human infections may be driven more by habitat overlap with the elective hosts species and specific risk factors, rather than a high intrinsic zoonotic potential. This perspective challenges the long-held assumptions regarding ORFV’s zoonotic threat, suggesting that transmission to humans could be a sporadic event rather than an evolutionary trait.

## Data Availability

Sequences obtained in the present study for the ORFV VIR gene were deposited in the GenBank database under the accession numbers OR338563–OR338593.

## Supplementary Materials

The following supporting information can be downloaded at: https://www.mdpi.com/article/10.3390/v18020222/s1, Figure S1: Map showing the geographical distribution of the Cuban samples analyzed in the present study; Figure S2: Phylogenetic signal analysis of the complete dataset. Likelihood mapping divides the equilateral triangle into seven distinct regions. The three corner trapezoids correspond to quartets that strongly support fully bifurcating, tree-like phylogenies. The side rectangles denote areas where the data do not clearly distinguish between two alternative tree topologies. The central region represents points P (posterior probabilities of the unrooted trees), where all three trees receive equal support; Figure S3. Principal Coordinates Analysis (PCoA) conducted on the whole dataset. The bi-dimensional plot displays the genetic differentiation among samples, by representing the number of nucleotide substitutions per site. Table S1: ORFV dataset reporting VIR gene sequences used in the present study. The table includes sample codes, GenBank accession numbers, and, where available, sequence origins, host species, collection dates, and corresponding references; Table S2: Sample sizes and genetic diversity estimates obtained for the VIR gene analyzed for host-associated populations. N: sample size; S: number of polymorphic sites; H: number of haplotypes; h: haplotype diversity; π: nucleotide diversity. Sites with gaps were not considered. Group subdivisions are explained within the text. Sequences from Sichuan takin and musk ox were represented by single individuals and were therefore not used for host-specific diversity estimates, although they were retained in the other analyses that included occasional hosts and all host populations.
