# Supplementary material for: Unravelling the Evolutionary Complexity of Orf Virus: A Global and Multi-Host Perspective
Source: Viruses. 2026 Feb 10;18(2):222. doi: 10.3390/v18020222 (PMC12945002; doi:10.3390/v18020222)
Supplement: Supplementary file 1 [file viruses-18-00222-s001.zip › Supplementary Materials Table S2.pdf]

Table S2. Sample sizes and genetic diversity estimates obtained for the VIR gene analyzed for host-associated populations. N: sample size; S: number of polymorphic sites; H: number of haplotypes; h: haplotype diversity; π: nucleotide diversity. Sites with gaps were not considered. Group subdivisions are explained within the text. Sequences from Sichuan takin and musk ox were represented by single individuals and were therefore not used for host-specific diversity estimates, although they were retained in the other analyses that included occasional hosts and all host populations.

**Species**	**N**	**S**	**H**	**h**	**π**
Goat	136	67	70	0.978	0.0361
Sheep	93	70	54	0.971	0.0366
Japanese-goat antelope	10	0	1	0	0
Small ruminant	6	24	2	0.333	0.0209
**All Ruminants**	247	35	43	0.931	0.0419
**Occasional hosts**	23	43	10	0.783	0.0266
Human	5	31	5	1	0.0377
**All host populations**	252	35	43	0.932	0.0418
