# Supplementary figures and images for: Unravelling the Evolutionary Complexity of Orf Virus: A Global and Multi-Host Perspective
Source: Viruses. 2026 Feb 10;18(2):222. doi: 10.3390/v18020222 (PMC12945002; doi:10.3390/v18020222)

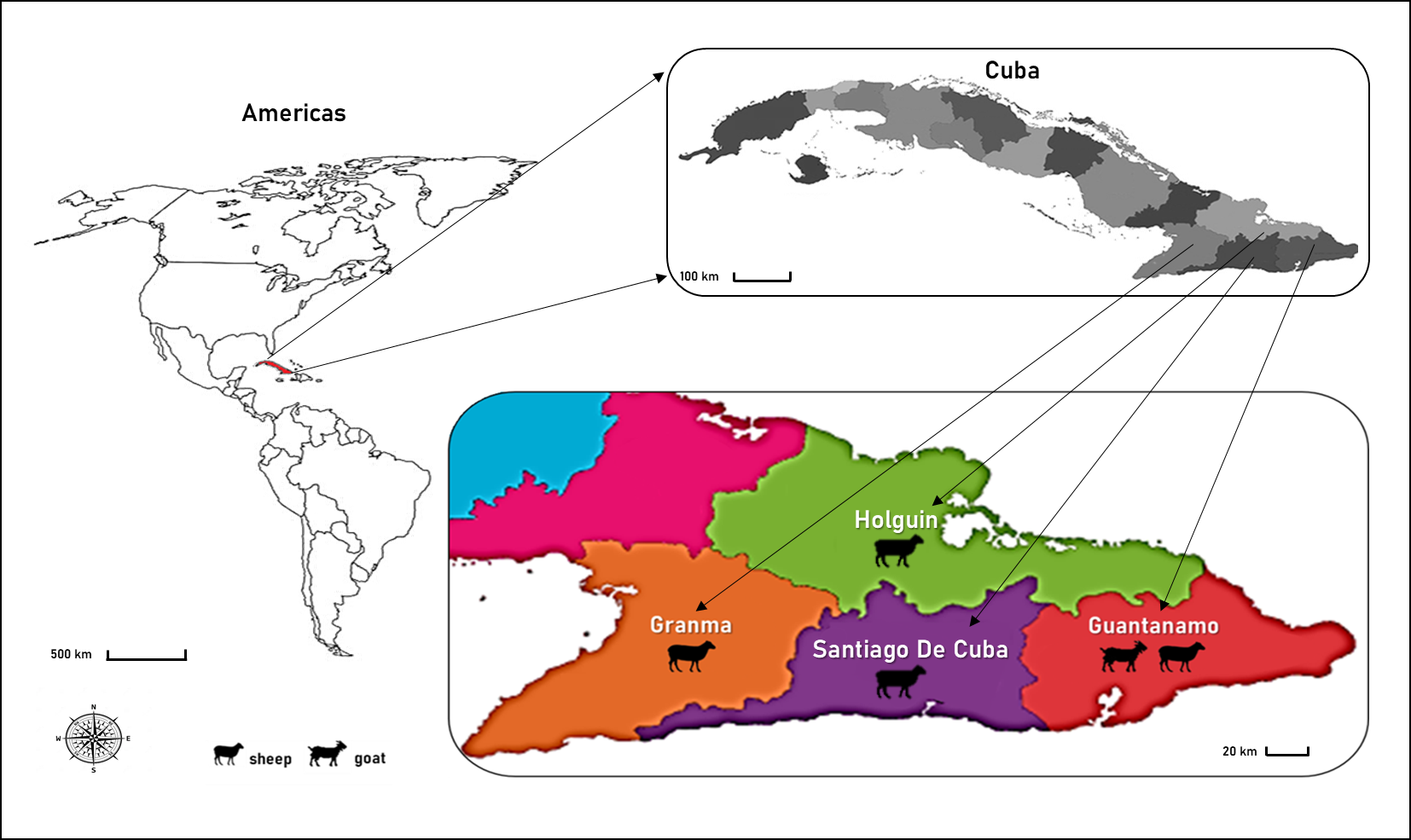

Supplement: Supplementary file 1 [file viruses-18-00222-s001.zip › Supplementary Figure S1.png]

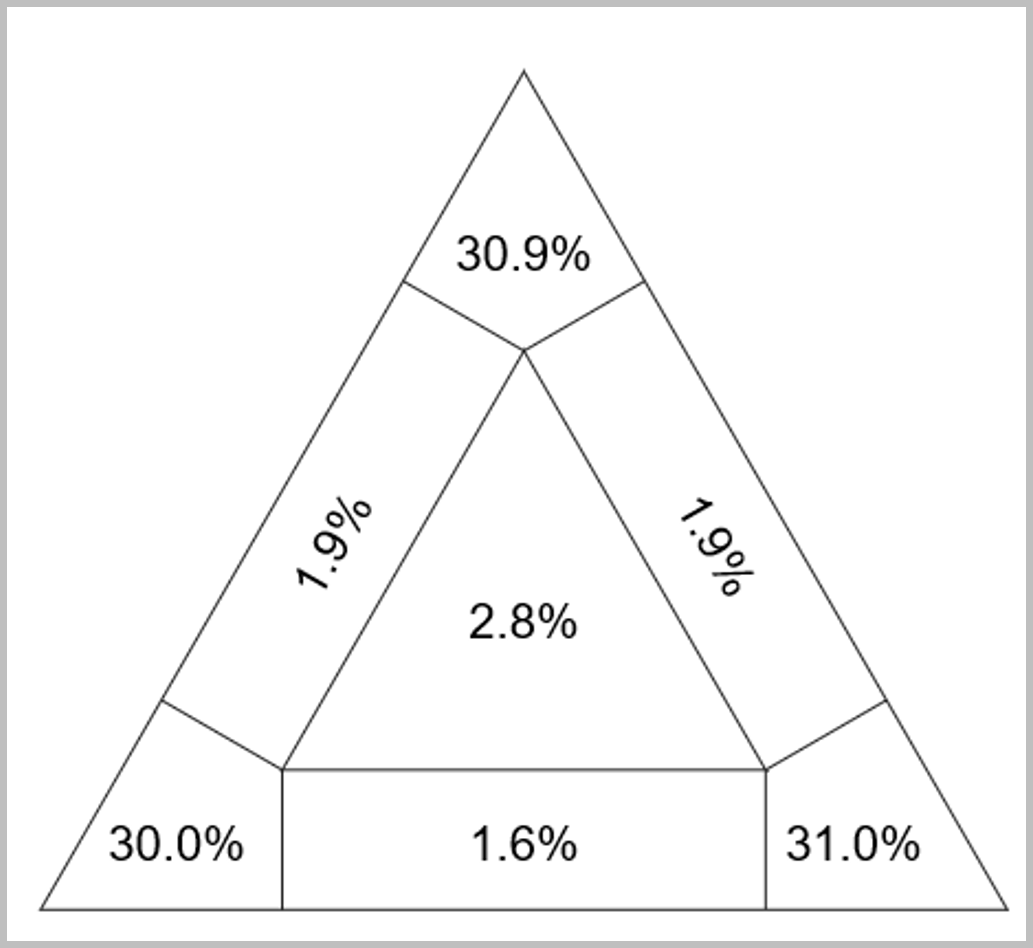

Supplement: Supplementary file 1 [file viruses-18-00222-s001.zip › Supplementary Figure S2.png]

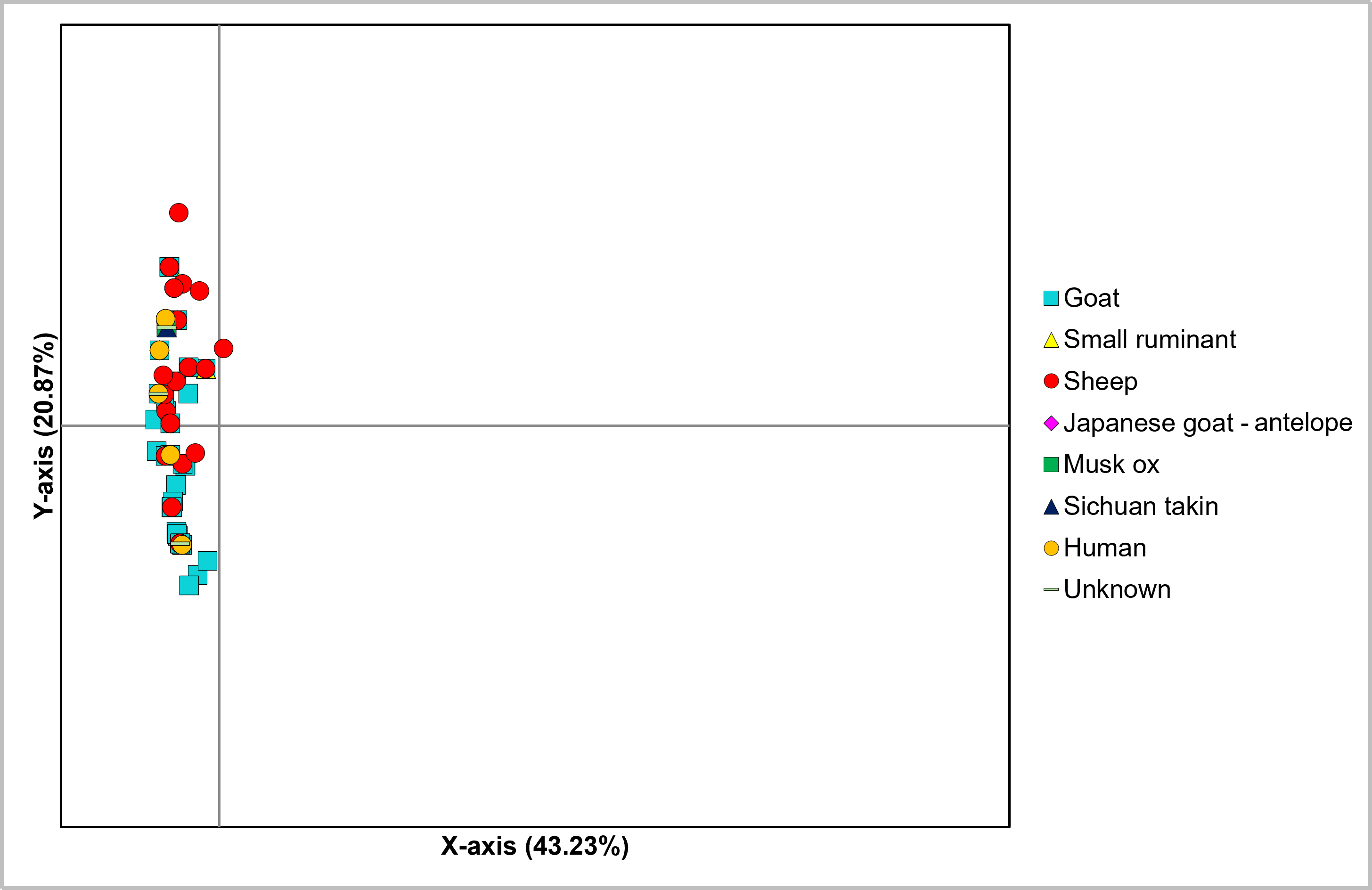

Supplement: Supplementary file 1 [file viruses-18-00222-s001.zip › Supplementary Figure S3.png]
